# Supplementary material for: Attenuation of the BOLD fMRI Signal and Changes in Functional Connectivity Affecting the Whole Brain in Presence of Brain Metastasis
Source: Cancers (Basel). 2024 May 25;16(11):2010. doi: 10.3390/cancers16112010 (PMC11171012; doi:10.3390/cancers16112010)
Supplement: Supplementary file 1 [file cancers-16-02010-s001.zip › cancers-3008147-supplementary.pdf]

Supplementary Material

1. Supplementary Tables

Table S1. Clusters of voxels functionally connected with seeds of the sensorimotor, default mode and salience networks for the patients with a left hemispheric metastasis and their matched controls for the paradigm of the left hand

Cluster	MNI (x;y;z)	size	size p-FDR	size p-unc	Regions
**Network: Sensorimotor network**					
Seed: Lateral (L)					
1	56;-26;26	952	<.001	<.001	PO (R); CO (R); INS (R); H (R); SGa (R); PT (R); POG (R); PP (R); TP (R); T1p (R); SGp (R)
2	12;18;30	412	<.001	<.001	CGa; PAC (R); PAC (L); JPL (L)
3	-60;-16;42	335	<.001	<.001	POG (L); PRG (L); SGa (L)
4	52;-14;40	244	.001	<.001	POG (R); PRG (R)
5	2;-12;68	198	.003	<.001	F1 (R); PRG (R); JPL (R); JPL (L); PRG (L)
6	-56;-20;22	130	.017	.002	CO (L); POG (L); PO (L); SGa (L), PT (L)
7	-50;38;0	113	.026	.003	FP (L); F3t (L)
8	-46;-34;64	107	.028	.004	POG (L); SPL (L)
9	-52;-2;0	101	.030	.005	PP (L); T1a (L); CO (L); PT (L)
Seed: Lateral (R)					
10	-50;-20;42	1518	<.001	<.001	POG (L); PO (L); PT (L); H (L); SGa (L); CO (L); T1a (L); PP (L); PRG (L); SGp (L); TO2 (L); OLi (L); OLs (L)
11	26;-60;0	500	<.001	<.001	LG (R); OF (R); CR 6 (R); CALC (R); TOF (R)
12	-38;-44;68	323	<.001	<.001	SPL (L); POG (L)
Seed: Superior					
13	-50;4;14	1847	<.001	<.001	POG (L); CO (L); PRG (L); PO (L); SGa (L); H (L); INS (L); SPL (L); PT (L); F3o (L)
14	56;-16;42	1337	<.001	<.001	CO (R); PO (R); POG (R); SGa (R); INS (R); H (R); PT (R); PRG (R)
15	16;-64;-52	303	<.001	<.001	CR 8 (R)
16	26;22;58	282	<.001	<.001	F1 (R); F2 (R)
17	-24;-60;-54	219	.001	<.001	CR 8 (L); CR 9 (L)
18	46;-52;54	176	.004	.001	AG (R); SGp (R); SPL (R); OLs (R)
19	10;18;58	114	.023	.003	F1 (R); F1 (L)
**Network: Default mode network**					
Seed: Medial prefrontal cortex					
20	-8;54;46	523	<.001	<.001	F1 (R); FP (L); FP (R); F1 (L)
21	-50;-22;-16	237	.003	<.001	T2p (L); T1p (L); T3p (L)
22	12;38;-20	176	.009	<.001	FP (R); FMC; FOC (R); SC
23	32;52;-8	162	.011	.001	FP (R)
24	-50;-72;40	120	.032	.003	OLs (L)
Seed: Lateral parietal (R)					
25	-48;-16;-2	257	.002	<.001	INS (L); PP (L); PUT (L); H (L); T1a (L); T1p (L); T2p (L); PT (L)
Seed: Posterior cingulate cortex					
26	0;-56;-30	390	<.001	<.001	CR 8 (L); VM 8; VM 4 5; VM 9; VM 6; CR 4 5 (L); CR 9 (L); VM 3; VM 10; CR 3 (L); VM 7
27	-6;16;40	341	<.001	<.001	CGa; PAC (L); JPL (R); PAC (R); F1 (R); JPL (L)

28	-46;-58;-4	240	.002	<.001	TOF (L); CR 6 (L); TO3 (L); OLi (L); TO2 (L)
29	18;-30;24	142	.019	.001	THL (R)
30	8;-54;-50	115	.037	.003	CR 9 (R); CR 8 (R)
**Network: Salience network**					
Seed: Anterior cingulate cortex					
31	-54;0;2	9876	<.001	<.001	OLs (L); CO (L); PO (L); PCN; CALC (L); OP (L); INS (L); CALC (R); OLi (L); CO (R); PT (L); INS (R); LG (L); OF (L); PRG (L); OF (R); TO2 (L); AG (L); SGp (L); H (L); CN (L); LG (R); TO2 (R); PO (R); PUT (L), POG (L); FO (R); PT (R); AG (R); FO (L); PP (L); H (R); FOC (L); T1a (L); SGa (L); T1p (R); OLi (R); T1p (L); TP (L); PRG (R); CN (R); CR 6 (R); SGp (R); OLs (R); CGp; SCLC (R); T2p (R); SCLC (L); PAL (L); F3o (L); PP (R); F3o (R); TP (R); T2p (L); TOF (R); TO3 (L); CR 6 (L); VM 4 5; VM 6
32	14;24;10	459	<.001	<.001	CAU (R)
33	16;-44;2	235	.002	<.001	CGp; PCN; LG (R); HP (R)
34	-20;2;28	148	.020	.001	CAU (L)
Seed: Anterior Insula (L)					
35	6;10;48	753	<.001	<.001	CGa; PAC (L); PAC (R); JPL (L); F1 (L); JPL (R); F1 (R)
36	34;0;46	365	<.001	<.001	PRG (R); F2 (R); F3o (R)
37	10;-36;50	315	<.001	<.001	PCN; CGp; POG (R); PRG (R); PRG (L); POG (L)
38	-32;34;38	205	.004	<.001	F2 (L); FP (L)
39	28;38;30	171	.009	.001	FP (R); F2 (R)
40	-48;0;40	140	.015	.002	PRG (L); F2 (L); F3o (L)
41	-54;-28;26	140	.015	.002	PO (L); SGa (L)
Seed: Anterior Insula (R)					
42	-4;4;46	397	<.001	<.001	PAC (R); CGa; JPL (L); JPL (R); PAC (L); F1 (R)
43	48;-46;14	221	.004	<.001	AG (R); SGp (R); TO2 (R)
44	30;34;28	130	.040	.002	F2 (R); FP (R)
45	-54;-20;24	120	.041	.003	PO (L); POG (L); SGa (L); CO (L)
Seed: Rostral prefrontal cortex (L)					
46	-48;-52;10	1081	<.001	<.001	TO2 (L); OLi (L); SGp (L); AG (L); OLs (L); T1p (L); PT (L); TO3 (L)
47	-40;2;8	638	<.001	<.001	CO (L); INS (L); PRG (L); FO (L); PP (L); T1a (L); F3o (L); FOC (L); TP (L)
48	-56;-42;28	379	<.001	<.001	PO (L); PT (L); SGp (L); H (L); SGa (L); CO (L)
49	6;46;46	242	.001	<.001	F1 (L); F1 (R); FP (R); PAC (L); FP (L); PAC (R)
50	30;-70;-52	197	.006	<.001	CR 8 (R); CR 7b (R); CRcr 2 (R)
51	-12;-56;58	182	.004	<.001	PCN; SPL (L); POG (L); OLs (L)
52	-6;-86;0	180	.004	<.001	CALC (L); OP (L); LG (L); OF (L)
53	26;-68;-22	178	.004	<.001	OF (R); CR 6 (R); TOF (R)
54	0;-32;-52	107	.032	.004	BS
Seed: Rostral prefrontal cortex (R)					
55	-4;48;42	1721	<.001	<.001	FP (L); F1 (L); FP (R); F1 (R); PAC (L); PAC (R); CGa
56	48;-50;6	1021	<.001	<.001	OF (R); OP (R); CR 6 (R); TO2 (R); OLi (R); CALC (R); LG (R); AG (R)
57	54;6;6	211	.005	<.001	INS (R); CO (R); PRG (R); FO (R); F3o (R); PUT (R)
Seed: Supramarginal gyrus (L)					
58	10;-4;60	293	.001	<.001	JPL (R); F1 (R); JPL (L); PRG (R)
59	-58;4;8	171	.014	.001	PRG (L); CO (L)

60	-34;-60;-44	148	.017	.001	CR 8 (L); CR 7b (L); CRcr 2 (L)
61	-46;-34;64	132	.017	.002	POG (L); SPL (L)
62	-26;-38;30	131	.017	.002	CGp; PCN
63	26;-70;-46	130	.017	.002	CR 8 (R); CR 7b (R); CRcr 2 (R)
Seed: Supramarginal gyrus (R)					
64	-54;-28;24	722	<.001	<.001	PO (L); SGa (L); PT (L); H (L); CO (L); POG (L); INS (L); SGp (L)
65	-60;2;6	415	<.001	<.001	CO (L); PRG (L); INS (L); PP (L); T1a (L); TP (L); FO (L)
66	-42;-40;64	291	<.001	<.001	SPL (L); POG (L); OLs (L)
67	-50;-50;8	162	.008	.001	TO2 (L); OLi (L); SGp (L)
68	-8;-16;70	118	.027	.003	PRG (L); JPL (L); F1 (L)

Abbreviations: MNI: Montreal Neurological Institute, FDR: false discovery rate, unc: uncorrected, L: left, R: right, AG: angular gyrus, AMYG: amygdala, BS: brain-stem, CAU: caudate, CALC: intracalcarine cortex, CGa: cingulate gyrus, anterior division, CGp: cingulate gyrus, posterior division, CN: cuneal cortex, CO: central opercular cortex, CR: cerebellum, CRcr: cerebellum crus, F1: superior frontal gyrus, F2: middle frontal gyrus, F3o: inferior frontal gyrus, pars opercularis, F3t: inferior frontal gyrus, pars triangularis, FMC: frontal medial cortex, FO: frontal operculum cortex, FOC: frontal orbital cortex, FP: frontal pole, H: Heschl's gyrus, HP: hippocampus, INS: insular cortex, JPL: juxtapositional lobule cortex, LG: lingual gyrus, OF: occipital fusiform gyrus, OLi: lateral occipital cortex, inferior division, OLs: lateral occipital cortex, superior division, OP: occipital pole, PAL: pallidum, PHa: parahippocampal gyrus, anterior division, PHp: parahippocampal gyrus, posterior division, PO: parietal operculum cortex, POG: postcentral gyrus, PP: planum polare, PRG: precentral gyrus, PT: planum temporale, PUT: putamen, SC: subcallosal cortex, SCLC: supracalcarine cortex, SGa: supramarginal gyrus, anterior division, SGp: supramarginal gyrus, posterior division, SPL: superior parietal lobule, T1a: superior temporal gyrus, anterior division, T1p: superior temporal gyrus, posterior division, T2a: middle temporal gyrus, anterior division, T2p: middle temporal gyrus, posterior division, T3a: inferior temporal gyrus, anterior division, T3p: inferior temporal gyrus, posterior division, TFa: temporal fusiform cortex, anterior division, TFp: temporal fusiform cortex, posterior division, THL: thalamus, TO2: middle temporal gyrus, temporooccipital part, TO3: inferior temporal gyrus, temporooccipital part, TOF: temporal occipital fusiform cortex, TP: temporal pole, VM: vermis

Table S2. Clusters of voxels functionally connected with seeds of the sensorimotor, default mode and salience networks for the patients with a left hemispheric metastasis and their matched controls for the paradigm of the right hand

Cluster	MNI (x;y;z)	size	size p-FDR	size p-unc	Regions
**Network: Sensorimotor network**					
Seed: Lateral (L)					
1	38;2;4	5233	<.001	<.001	PRG (R); POG (R); CO (R); INS (R); SGa (R); PUT (R); PP (R); H (R); PO (R); F3o (R); FO (R); F2 (R); PT (R); TP (R); SGp (R); F3t (R); PAL (R); AMYG (R); FOC (R)
2	-52;-2;0	2424	<.001	<.001	POG (L); PRG (L); CO (L); PP (L); INS (L); H (L); TP (L); PT (L); T1a (L); SGa (L); FO (L); PO (L); F3o (L); T1p (L); F2 (L)
3	60;-38;-16	1371	<.001	<.001	TO3 (R); OLi (R); TO2 (R); TOF (R); T3p (R); SGp (R); CR 6 (R); TFp (R); T2p (R); AG (R); CR 4 5 (R); OF (R)
4	-16;-66;-20	683	<.001	<.001	OF (L); CR 6 (L); CRcr 1 (L); TOF (L); CR 4 5 (L); LG (L); CR 8 (L); TFp (L)
5	-28;-56;16	291	<.001	<.001	
6	22;-56;-12	247	.001	<.001	OF (R); CR 6 (R); TOF (R); LG (R); CR 4 5 (R)
7	-18;-68;-50	205	.002	<.001	CR 8 (L); CR 7b (L)
8	-26;2;-8	172	.004	.001	PUT (L); PAL (L); INS (L)
9	16;-8;66	97	.043	.006	F1 (R); PRG (R)
Seed: Lateral (R)					
10	-50;-6;-2	3928	<.001	<.001	POG (L); PRG (L); CO (L); INS (L); PUT (L); PT (L); PO (L); H (L); PP (L); SGa (L); SGp (L); T1a (L); FO (L); TP (L); PAL (L); AG (L); T1p (L); FOC (L); F3o (L); CAU (L)

11	52;2;4	1949	<.001	<.001	PRG (R); CO (R); INS (R); PP (R); POG (R); PUT (R); FO (R); H (R); TP (R); PAL (R); AMYG (R); F3o (R); SGa (R); PO (R); PT (R); F2 (R); F3t (R)
12	-12;-70;-14	828	<.001	<.001	CR 6 (L); OF (L); LG (L); OLi (L); CALC (L); CRcr 1 (L); TOF (L); CR 4 5 (L)
13	46;-56;-18	486	<.001	<.001	TO3 (R); OLi (R); TOF (R); TO2 (R); TFp (R)
14	22;-64;-52	230	.002	<.001	CR 8 (R); CR 9 (R)
15	-16;-66;-50	222	.002	<.001	CR 8 (L); CR 7b (L)
16	-40;-58;-26	158	.010	.001	TOF (L); CRcr 1 (L); CR 6 (L); TO3 (L)
17	22;-56;-10	121	.028	.003	CR 6 (R); LG (R); CR 4 5 (R); TOF (R)
18	-14;0;70	111	.035	.004	F1 (L)
Seed: Superior					
19	50;-4;4	1231	<.001	<.001	CO (R); INS (R); PP (R); H (R); PRG (R); PUT (R); PO (R); TP (R); PT (R); F3o (R); FO (R); T1a (R)
20	28;-58;-52	471	<.001	<.001	CR 8 (R); CR 7b (R); CRcr 2 (R); CR 9 (R)
21	-42;-54;-20	217	.004	<.001	TOF (L); CRcr 1 (L); TO3 (L); CR 6 (L)
22	34;-50;-22	197	.006	<.001	CR 6 (R); TOF (R); OF (R); CR 4 5 (R); CRcr 1 (R)
23	-54;-2;4	184	.007	.001	CO (L); PP (L); PRG (L); TP (L); INS (L)
24	14;-22;6	162	.010	.001	THL (R); HP (R)
25	6;48;50	138	.018	.002	FP (R); F1 (R); F1 (L)
26	-20;-70;-48	131	.019	.002	CR 8 (L); CR 7b (L)
27	-20;-56;-52	128	.019	.003	CR 8 (L); CR 9 (L)
28	-44;-76;-12	124	.019	.003	OLi (L); OF (L)
29	-50;-30;30	117	.021	.004	PO (L); SGa (L); POG (L)
30	-40;46;26	99	.036	.007	FP (L); F2 (L)
**Network: Default mode network**					
Seed: Medial prefrontal cortex					
31	-46;-68;32	475	<.001	<.001	OLs (L); AG (L)
32	38;54;-4	418	<.001	<.001	FP (R)
33	-16;34;-30	321	<.001	<.001	FOC (L); TP (L); FP (L); INS (L)
34	-48;42;4	234	.002	<.001	FP (L); F3t (L)
35	-6;66;10	214	.003	<.001	FP (L); FP (R)
36	-4;-54;32	200	.004	<.001	PCN; CGp
37	48;2;20	108	.046	.004	PRG (R); CO (R); F3o (R)
38	-22;-76;-56	107	.046	.004	CR 8 (L); CR 7b (L)
Seed: Lateral parietal (L)					
39	-28;18;40	564	<.001	<.001	F2 (L); F1 (L)
40	-14;64;10	291	.001	<.001	FP (L); FP (R); PAC (L); FMC
41	38;-74;-40	209	.004	<.001	CRcr 2 (R); CRcr 1 (R)
42	-32;36;-16	124	.031	.003	FOC (L); FP (L)
43	14;28;-20	120	.031	.003	FP (R); FOC (R); SC
44	-2;-56;-48	108	.038	.005	CR 9 (R); CR 9 (L)
Seed: Lateral parietal (R)					
45	16;54;22	176	.030	.001	FP (R); F1 (R); PAC (R)
46	34;30;-10	159	.030	.001	FOC (R); TP (R); FP (R)
Seed: Posterior cingulate cortex					

47	-2;-58;-46	342	<.001	<.001	CR 9 (R); CR 9 (L); CR 8 (R); CR 8 (L); VM 9; VM 8; BS
**Network: Salience network**					
Seed: Anterior cingulate cortex					
48	42;12;-4	3546	<.001	<.001	INS (R); CO (R); AG (R); TO2 (R); SGp (R); OLi (R); PO (R); TP (R); FO (R); PRG (R); H (R); F3o (R); PT (R); PP (R); T1p (R); PUT (R); FOC (R); SGa (R); TO3 (R); T2p (R); T1a (R); OLs (R); PAL (R); POG (R); F3t (R); T2a (R)
49	-40;10;-2	2608	<.001	<.001	INS (L); PUT (L); CO (L); PO (L); H (L); PT (L); PP (L); FO (L); PRG (L); THL (L); F3o (L); T1a (L); TP (L); SGa (L); PAL (L); T1p (L); FOC (L); POG (L); HP (L)
50	12;-66;-40	488	<.001	<.001	CR 8 (R); VM 8; CR 7b (R); CR 9 (R); CRcr 2 (R); CRcr 1 (R)
51	4;-60;0	348	<.001	<.001	CALC (L); LG (L); LG (R); PCN; VM 4 5; SCLC (L); SCLC (R)
52	12;-88;14	309	<.001	<.001	OP (R); CALC (R); CN (R); CN (L)
53	32;-48;-30	282	.001	<.001	CR 6 (R); CR 4 5 (R); BS; TFp (R); PHp (R); TOF (R); CRcr 1 (R)
54	18;-72;-2	275	.001	<.001	LG (R); CR 6 (R); OF (R); CALC (R); CR 4 5 (R); TOF (R)
55	-62;-58;10	213	.002	<.001	AG (L); TO2 (L); SGp (L); OLi (L)
56	-42;-70;8	133	.021	.002	OLi (L)
57	-18;-58;52	124	.025	.003	OLs (L); SPL (L); PCN
58	-14;-72;-48	112	.034	.004	CR 8 (L); CR 7b (L)
59	-6;-24;6	100	.047	.006	THL (L); BS
60	-2;18;62	96	.049	.007	F1 (R); F1 (L); JPL (R); JPL (L)
Seed: Anterior Insula (L)					
61	-54;4;40	2378	<.001	<.001	PRG (L); F2 (L); FP (L); F3t (L); F3o (L)
62	38;36;42	2105	<.001	<.001	F2 (R); PRG (R); F3o (R); FP (R); INS (R); CO (R); F3t (R); PUT (R)
63	-24;-52;54	1914	<.001	<.001	POG (L); SPL (L); SGa (L); OLs (L); PO (L); CO (L); SGp (L); PT (L); PCN
64	-6;8;54	1759	<.001	<.001	JPL (L); F1 (R); PAC (L); F1 (L); PAC (R); CGa; PRG (R); JPL (R); PRG (L); POG (L); F2 (R); PCN
65	50;-18;44	1643	<.001	<.001	POG (R); SGa (R); SGp (R); PO (R); SPL (R); PRG (R); PT (R)
66	-42;-46;-30	874	<.001	<.001	CR 6 (L); CRcr 1 (L); OF (L); TOF (L); CR 8 (L); CR 4 5 (L); TFp (L); OLi (L); TO3 (L)
67	-22;-36;28	580	<.001	<.001	
68	-20;-72;-48	396	<.001	<.001	CR 8 (L); CR 7b (L); CRcr 2 (L)
69	54;-52;12	280	<.001	<.001	TO2 (R); AG (R); SGp (R); OLi (R)
70	-48;-4;-2	231	.001	<.001	PP (L); F3o (L); H (L); CO (L); TP (L); INS (L); FO (L); PRG (L)
71	16;-66;-44	226	.001	<.001	CR 8 (R); CR 7b (R); CR 6 (R)
72	30;-68;-16	210	.001	<.001	CR 6 (R); OF (R); TOF (R)
73	-60;-52;16	131	.010	.002	AG (L); TO2 (L); SGp (L)
74	-26;-16;18	125	.012	.002	THL (L); CAU (L); INS (L); PUT (L)
75	34;-52;16	118	.014	.002	
76	-22;4;4	88	.038	.007	PUT (L); PAL (L)
Seed: Anterior Insula (R)					
77	-30;-36;54	6335	<.001	<.001	POG (L); SPL (L); PRG (L); PCN; SGa (L); SGp (L); OLs (L); OLs (R); CO (L); CGp; AG (L); PO (L); H (L); SPL (R); TO2 (L); INS (L); JPL (L); PRG (R); POG (R); PT (L); F2 (L); PP (L)
78	44;4;42	4319	<.001	<.001	PRG (R); SGp (R); POG (R); SGa (R); F2 (R); AG (R); SPL (R); TO2 (R); F3o (R); PO (R); CO (R); OLi (R); PT (R); PP (R)
79	4;12;42	1630	<.001	<.001	F1 (R); PAC (R); CGa; JPL (R); JPL (L); PAC (L); F1 (L); F2 (R); PRG (R)

80	-40;-48;-34	1257	<.001	<.001	CR 6 (L); CRcr 1 (L); OF (L); TOF (L); TFp (L); LG (L); TO3 (L); OLi (L); CR 4 5 (L); CR 8 (L)
81	34;38;38	934	<.001	<.001	FP (R); F2 (R)
82	22;-64;-56	414	<.001	<.001	CR 8 (R); VM 8; CR 7b (R); CRcr 2 (R)
83	-18;-72;-50	271	<.001	<.001	CR 8 (L); CR 7b (L); CRcr 2 (L)
84	26;-86;24	265	<.001	<.001	OLs (R); OP (R); CN (R)
85	-44;44;28	211	.001	<.001	FP (L); F2 (L)
86	-46;-70;2	172	.002	<.001	OLi (L)
87	-10;-18;68	148	.005	.001	F1 (L); PRG (L)
88	48;-62;-6	103	.020	.0037	OLi (R); TO3 (R)
89	-24;-50;14	85	.033	.007	
90	32;-52;16	85	.033	.007	
91	20;36;-6	79	.039	.009	
Seed: Rostral prefrontal cortex (L)					
92	-38;8;10	865	<.001	<.001	CO (L); PRG (L); INS (L); PUT (L); FO (L); THL (L); PP (L); F3o (L); PAL (L); T1a (L); HP (L); TP (L)
93	-14;-62;48	820	<.001	<.001	SPL (L); POG (L); OLs (L); PCN; SGa (L); SGp (L)
94	2;52;40	774	<.001	<.001	FP (R); F1 (R); F1 (L); FP (L); PAC (R)
95	50;2;0	601	<.001	<.001	CO (R); INS (R); PRG (R); F3o (R); PP (R); TP (R); FO (R); PT (R); T1a (R)
96	-40;-50;-34	441	<.001	<.001	CRcr 1 (L); TFp (L); TOF (L); CR 6 (L); CR 8 (L); T3p (L); CR 4 5 (L); TO3 (L)
97	-52;-28;24	355	<.001	<.001	PO (L); SGa (L); CO (L); PT (L); POG (L); SGp (L)
98	16;-64;4	313	<.001	<.001	CALC (R); LG (R); LG (L); VM 4 5; PCN; CALC (L)
99	28;-38;-22	252	.001	<.001	CR 4 5 (R); TOF (R); TFp (R); CR 6 (R); VM 4 5; PHp (R); LG (R)
100	16;-68;-44	230	.001	<.001	CR 8 (R); CRcr 2 (R); CR 7b (R)
101	-12;-70;-36	225	.001	<.001	CR 7b (L); CR 8 (L); CRcr 2 (L); CRcr 1 (L); CR 6 (L)
102	42;-32;16	186	.003	<.001	PO (R); PT (R); H (R); INS (R)
103	22;-70;-14	125	.019	.003	OF (R); CR 6 (R); LG (R)
104	-6;-54;64	116	.023	.004	PCN; SPL (L); OLs (L)
105	-18;-62;8	105	.030	.005	CALC (L); LG (L); PCN
106	0;-96;22	99	.035	.006	OP (L); OP (R); CN (L); CN (R)
107	-18;34;24	95	.037	.007	PAC (L)
108	-58;-54;8	90	.042	.009	TO2 (L); AG (L)
Seed: Rostral prefrontal cortex (R)					
109	44;10;-4	2280	<.001	<.001	INS (R); CO (R); FO (R); PUT (R); TP (R); F3o (R); PRG (R); THL (R); PP (R); PAL (R); F3t (R); T1a (R); FOC (R); H (R)
110	54;-50;20	1116	<.001	<.001	SGp (R); AG (R); SGa (R); POG (R); TO2 (R); PO (R); SPL (R)
111	-18;-70;-48	848	<.001	<.001	CR 8 (R); CR 8 (L); CR 7b (L); VM 8; CRcr 2 (L); CR 7b (R); CRcr 2 (R)
112	46;-56;-8	373	<.001	<.001	OLi (R); TO3 (R); TOF (R); OLs (R)
113	18;2;64	275	.001	<.001	F1 (R); JPL (R); PRG (R)
114	-42;-32;66	257	.001	<.001	POG (L)
115	-42;10;0	210	.003	<.001	INS (L); CO (L); FO (L); PP (L)
116	30;-36;-30	200	.003	<.001	CR 6 (R); CR 4 5 (R); TFp (R); PHp (R); CRcr 1 (R); CR 10 (R)
117	-42;-66;4	195	.003	<.001	OLi (L); TO2 (L)
118	52;-18;16	185	.004	<.001	H (R); CO (R); T1p (R); PO (R); T2p (R); PT (R)
119	8;-68;48	160	.008	.001	PCN; OLs (R)

120	-68;-18;24	109	.036	.004	POG (L); T1p (L); SGa (L)
121	38;40;44	99	.046	.006	FP (R)
Seed: Supramarginal gyrus (L)					
122	22;-72;-50	680	<.001	<.001	CR 8 (R); CR 6 (R); CR 7b (R); TOF (R); CRcr 2 (R); CR 4 5 (R); CR 9 (R); CRcr 1 (R)
123	66;-32;40	676	<.001	<.001	SGa (R); POG (R); SGp (R); PO (R)
124	-60;-62;0	512	<.001	<.001	TO2 (L); OLi (L); AG (L); SGp (L)
125	-26;-4;-12	469	<.001	<.001	INS (L); CO (L); PP (L); PUT (L); TP (L); AMYG (L); FO (L); T1a (L)
126	-20;-42;22	410	<.001	<.001	
127	8;2;62	383	<.001	<.001	JPL (R); CGa; JPL (L); F1 (R); PAC (L); F1 (L)
128	-44;-50;-20	294	<.001	<.001	TOF (L); CRcr 1 (L); TO3 (L); TFp (L); CR 6 (L)
129	46;4;-2	208	.003	<.001	INS (R); CO (R); F3o (R); TP (R); PP (R); PRG (R); FO (R)
130	32;-54;18	195	.004	<.001	
131	-26;-78;-52	156	.008	.001	CR 7b (L); CR 8 (L); CRcr 2 (L)
132	-48;34;10	155	.008	.001	F3t (L); FP (L); F2 (L)
133	62;-64;2	154	.008	.001	TO2 (R); OLi (R); AG (R)
134	56;10;20	152	.008	.001	PRG (R); F3o (R)
135	14;16;2	116	.023	.004	CAU (R)
136	42;-60;42	93	.046	.009	OLs (R); AG (R)
Seed: Supramarginal gyrus (R)					
137	-52;0;-2	2149	<.001	<.001	CO (L); INS (L); PUT (L); PO (L); PRG (L); PP (L); SGa (L); H (L); POG (L); SGp (L); TP (L); PT (L); PAL (L); T1a (L); FO (L)
138	4;0;62	1252	<.001	<.001	JPL (L); CGa; JPL (R); F1 (L); F1 (R); PAC (L); PAC (R)
139	-32;-34;64	1212	<.001	<.001	POG (L); PRG (L); SPL (L); SGa (L); OLs (L); F2 (L)
140	24;-66;-48	1061	<.001	<.001	CR 8 (R); CR 8 (L); CR 7b (L); CR 7b (R); VM 8; CRcr 2 (L); CR 9 (R); CRcr 2 (R)
141	40;8;-4	899	<.001	<.001	INS (R); CO (R); PRG (R); PUT (R); FO (R); TP (R); F3o (R); PP (R); PAL (R)
142	-40;-50;-32	466	<.001	<.001	CRcr 1 (L); CR 6 (L); TOF (L); OF (L); TFp (L); TO3 (L)
143	54;0;50	393	<.001	<.001	PRG (R); POG (R); F2 (R)
144	38;-54;34	381	<.001	<.001	OLs (R); AG (R)
145	-46;-66;8	147	.011	.001	OLi (L); TO2 (L)
146	-60;-58;8	131	.016	.002	TO2 (L); AG (L); SGp (L)
147	32;-56;16	128	.016	.002	
148	38;38;42	124	.017	.002	FP (R); F2 (R)
149	16;12;8	118	.019	.003	CAU (R); PUT (R)
150	64;-56;16	113	.020	.003	AG (R); TO2 (R); SGp (R)

Abbreviations: MNI: Montreal Neurological Institute, FDR: false discovery rate, unc: uncorrected, L: left, R: right, AG: angular gyrus, AMYG: amygdala, BS: brain-stem, CAU: caudate, CALC: intracalcarine cortex, CGa: cingulate gyrus, anterior division, CGp: cingulate gyrus, posterior division, CN: cuneal cortex, CO: central opercular cortex, CR: cerebellum, CRcr: cerebellum crus, F1: superior frontal gyrus, F2: middle frontal gyrus, F3o: inferior frontal gyrus, pars opercularis, F3t: inferior frontal gyrus, pars triangularis, FMC: frontal medial cortex, FO: frontal operculum cortex, FOC: frontal orbital cortex, FP: frontal pole, H: Heschl's gyrus, HP: hippocampus, INS: insular cortex, JPL: juxtapositional lobule cortex, LG: lingual gyrus, OF: occipital fusiform gyrus, OLi: lateral occipital cortex, inferior division, OLs: lateral occipital cortex, superior division, OP: occipital pole, PAL: pallidum, PHa: parahippocampal gyrus, anterior division, PHp: parahippocampal gyrus, posterior division, PO: parietal operculum cortex, POG: postcentral gyrus, PP: planum polare, PRG: precentral gyrus, PT: planum temporale, PUT: putamen, SC: subcallosal cortex, SCLC: supracalcarine cortex, SGa: supramarginal gyrus, anterior division, SGp: supramarginal gyrus, posterior division, SPL: superior parietal lobule, T1a: superior temporal gyrus, anterior division, T1p: superior temporal gyrus, posterior division, T2a: middle temporal gyrus, anterior division, T2p: middle temporal gyrus, posterior division, T3a: inferior temporal gyrus, anterior division, T3p: inferior temporal gyrus, posterior division, TFa: temporal

fusiform cortex, anterior division, TFp: temporal fusiform cortex, posterior division, THL: thalamus, TO2: middle temporal gyrus, temporooccipital part, TO3: inferior temporal gyrus, temporooccipital part, TOF: temporal occipital fusiform cortex, TP: temporal pole, VM: vermis

Table S3. Clusters of voxels functionally connected with seeds of the sensorimotor, default mode and salience networks for the patients with a left hemispheric metastasis and their matched controls for the paradigm of the left foot

Cluster	MNI (x;y;z)	size	size p-FDR	size p-unc	Regions
**Network: Sensorimotor network**					
Seed: Lateral (L)					
1	-4;58;40	327	<.001	<.001	FP (L); F1 (R); F1 (L); FP (R)
2	-24;2;74	249	.001	<.001	F1 (L); PRG (L)
3	-64;-18;14	101	.042	.004	CO (L); POG (L); PT (L); PO (L); SGa (L)
Seed: Lateral (R)					
4	-14;-6;76	165	.016	<.001	F1 (L); PRG (L)
Seed: Superior					
5	0;42;46	812	<.001	<.001	F1 (R); F1 (L); FP (R); FP (L); PAC (R); PAC (L)
6	50;-30;36	301	<.001	<.001	SGa (R); PO (R); POG (R)
7	48;0;2	204	.002	<.001	CO (R); INS (R); PP (R); H (R); PRG (R)
8	-42;-6;0	185	.002	<.001	INS (L); CO (L); PP (L); TP (L); PRG (L)
9	-56;-24;10	173	.006	<.001	PT (L); SGa (L); CO (L); PO (L); H (L)
10	-34;12;54	125	.010	.001	F2 (L)
11	-18;-38;78	88	.035	.006	POG (L)
**Network: Default mode network**					
Seed: Medial prefrontal cortex					
12	-50;-70;34	454	<.001	<.001	OLs (L); AG (L)
Seed: Lateral parietal (L)					
13	-8;-94;28	213	.004	<.001	OP (L); CN (R); OLs (L); CN (L); OP (R)
14	-24;20;52	165	.008	<.001	F1 (L); F2 (L)
15	10;54;16	110	.034	.003	PAC (R); FP (R); F1 (R)
**Network: Salience network**					
Seed: Anterior cingulate cortex					
16	10;40;38	950	<.001	<.001	FP (L); F1 (R); F1 (L); FP (R); PAC (R); PAC (L)
17	-10;2;76	416	<.001	<.001	F1 (L); PRG (L)
18	60;-44;18	315	<.001	<.001	SGp (R); AG (R); TO2 (R); PT (R); PO (R)
19	42;14;2	251	.001	<.001	FO (R); INS (R); CO (R); PP (R); TP (R); PRG (R); F3o (R)
20	-22;-92;10	180	.003	<.001	OP (L); OLs (L)
21	16;-62;6	149	.007	.001	CALC (R); CN (R); SCLC (R); LG (R); PCN
22	26;4;72	142	.008	.001	F1 (R); PRG (R)
23	46;-20;0	115	.017	.002	H (R); T1p (R); PP (R); T2p (R)
24	20;10;38	111	.017	.003	
25	24;-86;-28	102	.022	.004	CRcr 1 (R); CRcr 2 (R)
26	48;-64;-6	97	.024	.004	OLi (R); TO2 (R); TO3 (R)
27	50;-32;34	76	.049	.010	PO (R); PT (R)
Seed: Anterior Insula (L)					

28	-50;0;42	313	<.001	<.001	PRG (L)
29	-36;36;26	138	.017	.001	F2 (L); FP (L)
30	50;-38;54	133	.017	.001	SGp (R); SPL (R); SGa (R); POG (R)
31	-38;-44;60	130	.017	.001	SPL (L); POG (L)
Seed: Anterior Insula (R)					
32	50;-38;52	366	<.001	<.001	SGp (R); SGa (R); SPL (R); AG (R)
33	-46;4;24	220	.001	<.001	PRG (L); F3o (L)
34	-36;34;26	123	.022	.001	FP (L); F2 (L)
35	-58;-26;38	107	.029	.002	SGa (L); POG (L)
Seed: Rostral prefrontal cortex (L)					
36	-50;0;8	320	<.001	<.001	CO (L); INS (L); TP (L); PP (L); FO (L); PRG (L); F3o (L)
37	50;6;-2	295	<.001	<.001	CO (R); PP (R); H (R); TP (R); INS (R); FO (R); PRG (R); F3o (R); T1a (R)
38	0;46;50	294	<.001	<.001	F1 (R); FP (L); F1 (L); FP (R)
39	-58;-68;-2	214	.001	<.001	TO2 (L); OLi (L); SGp (L); AG (L)
40	-2;62;20	188	.001	<.001	FP (L); FP (R); F1 (L); PAC (R); PAC (L)
41	-32;-14;74	122	.010	.001	PRG (L); F1 (L)
42	-62;-28;16	83	.034	.007	SGa (L); PO (L); PT (L); POG (L)
43	16;-64;12	83	.034	.007	CALC (R); PCN; SCLC (R)
Seed: Rostral prefrontal cortex (R)					
44	58;-60;2	210	.003	<.001	TO2 (R); AG (R); OLi (R)
45	-6;64;22	120	.025	.002	FP (L); F1 (L)
Seed: Supramarginal gyrus (L)					
46	-38;-46;66	204	.005	<.001	POG (L); SPL (L)
47	-46;-66;38	118	.039	.002	OLs (L)
48	-52;0;0	100	.049	.004	CO (L); PP (L); TP (L); PRG (L); F3o (L); FO (L)
Seed: Supramarginal gyrus (R)					
49	-52;8;-6	190	.005	<.001	CO (L); TP (L); PP (L); INS (L); FO (L); PRG (L); F3o (L)
50	-10;-26;66	139	.013	.001	PRG (L); JPL (L); F1 (L); POG (L); PRG (R)

Abbreviations: MNI: Montreal Neurological Institute, FDR: false discovery rate, unc: uncorrected, L: left, R: right, AG: angular gyrus, AMYG: amygdala, BS: brain-stem, CAU: caudate, CALC: intracalcarine cortex, CGa: cingulate gyrus, anterior division, CGp: cingulate gyrus, posterior division, CN: cuneal cortex, CO: central opercular cortex, CR: cerebellum, CRcr: cerebellum crus, F1: superior frontal gyrus, F2: middle frontal gyrus, F3o: inferior frontal gyrus, pars opercularis, F3t: inferior frontal gyrus, pars triangularis, FMC: frontal medial cortex, FO: frontal operculum cortex, FOC: frontal orbital cortex, FP: frontal pole, H: Heschl's gyrus, HP: hippocampus, INS: insular cortex, JPL: juxtapositional lobule cortex, LG: lingual gyrus, OF: occipital fusiform gyrus, OLi: lateral occipital cortex, inferior division, OLs: lateral occipital cortex, superior division, OP: occipital pole, PAL: pallidum, PHa: parahippocampal gyrus, anterior division, PHp: parahippocampal gyrus, posterior division, PO: parietal operculum cortex, POG: postcentral gyrus, PP: planum polare, PRG: precentral gyrus, PT: planum temporale, PUT: putamen, SC: subcallosal cortex, SCLC: supracalcarine cortex, SGa: supramarginal gyrus, anterior division, SGp: supramarginal gyrus, posterior division, SPL: superior parietal lobule, T1a: superior temporal gyrus, anterior division, T1p: superior temporal gyrus, posterior division, T2a: middle temporal gyrus, anterior division, T2p: middle temporal gyrus, posterior division, T3a: inferior temporal gyrus, anterior division, T3p: inferior temporal gyrus, posterior division, TFa: temporal fusiform cortex, anterior division, TFp: temporal fusiform cortex, posterior division, THL: thalamus, TO2: middle temporal gyrus, temporooccipital part, TO3: inferior temporal gyrus, temporooccipital part, TOF: temporal occipital fusiform cortex, TP: temporal pole, VM: vermis

Table S4. Clusters of voxels functionally connected with seeds of the sensorimotor, default mode and salience networks for the patients with a left hemispheric metastasis and their matched controls for the paradigm of the right foot

Cluster	MNI (x;y;z)	size	size p-FDR	size p-unc	Regions

**Network: Sensorimotor network**					
Seed: Lateral (L)					
1	-62;-54;6	199	.002	<.001	TO2 (L); OLi (L); SGp (L); AG (L)
2	-38;-14;66	197	.002	<.001	PRG (L)
3	66;-40;22	94	.046	.004	SGp (R); PO (R); SGa (R)
4	-50;-8;14	88	.046	.005	CO (L); H (L)
5	44;-6;64	83	.046	.006	PRG (R); POG (R)
Seed: Lateral (R)					
6	-60;-4;2	175	.014	<.001	CO (L); H (L); PP (L); T1a (L); INS (L)
Seed: Superior					
7	10;18;54	3157	<.001	<.001	F1 (R); F1 (L); F2 (L); PAC (R); PAC (L); F2 (R); FP (R); PRG (L); JPL (R); JPL (L); PRG (R)
8	42;-30;26	844	<.001	<.001	PO (R); POG (R); SGp (R); SGa (R); CO (R); AG (R); PT (R); T1p (R)
9	-58;-26;22	244	.001	<.001	PO (L); SGa (L); CO (L); POG (L); INS (L)
10	40;20;44	158	.006	.001	F2 (R); F3o (R)
11	8;-60;-2	117	.018	.002	LG (R); VM 4 5; LG (L); CALC (R); VM 6
12	20;-66;-54	101	.027	.004	CR 8 (R)
13	62;-2;-2	81	.046	.008	CO (R); T1a (R); PP (R); INS (R)
14	-50;-4;4	80	.046	.008	CO (L); INS (L); PP (L)
**Network: Default mode network**					
Seed: Medial prefrontal cortex					
15	-40;48;2	279	<.001	<.001	FP (L); FOC (L)
16	36;24;2	241	.001	<.001	FO (R); F3o (R); INS (R); F3t (R); FOC (R); PUT (R)
17	32;52;4	232	.001	<.001	FP (R)
18	0;66;4	121	.013	.002	FP (R); FP (L)
Seed: Lateral parietal (L)					
19	-34;-50;10	134	.018	.001	
20	-24;20;58	123	.018	.002	F1 (L); F2 (L)
Seed: Lateral parietal (R)					
21	24;32;38	154	.016	.001	F2 (R); F1 (R); FP (R)
**Network: Salience network**					
Seed: Anterior cingulate cortex					
22	-38;-32;20	2949	<.001	<.001	INS (L); CO (L); PO (L); AG (L); TO2 (L); PT (L); SGp (L); H (L); T1p (L); OLs (L); FOC (L); OLi (L); FO (L); SGa (L); T2p (L); PP (L); PRG (L); F3o (L); POG (L); TP (L); AMYG (L)
23	30;-30;20	1080	<.001	<.001	TO2 (R); SGp (R); PO (R); AG (R); PT (R); INS (R); H (R); CO (R); OLi (R); SGa (R); PUT (R); T1p (R); POG (R)
24	18;20;42	837	<.001	<.001	F1 (R); FP (R); PAC (R); F2 (R); CAU (R)
25	36;2;-14	836	<.001	<.001	INS (R); TP (R); FOC (R); FO (R); CO (R); F3o (R); FP (R); PUT (R); F3t (R); PP (R)
26	12;-86;22	609	<.001	<.001	CN (R); CALC (R); CN (L); OP (L); OLs (L); CALC (L); PCN; SCLC (R); SCLC (L); OP (R)
Seed: Anterior Insula (L)					
27	-50;2;40	388	<.001	<.001	PRG (L); F2 (L)
28	-2;16;46	272	<.001	<.001	PAC (L); CGa; PAC (R); F1 (L); F1 (R); JPL (L)
29	-44;-2;56	177	.003	<.001	PRG (L); F2 (L)

30	-24;-46;16	142	.008	.001	
31	-38;40;30	117	.015	.002	F2 (L); FP (L)
Seed: Anterior Insula (R)					
32	2;-2;40	1659	<.001	<.001	JPL (R); CGa; JPL (L); PAC (R); F1 (R); PRG (L); PAC (L); F1 (L); PRG (R); CGp; POG (L)
33	54;-24;42	774	<.001	<.001	SGa (R); POG (R); SGp (R); PRG (R)
34	60;12;22	445	<.001	<.001	PRG (R); F3o (R); F2 (R)
35	32;38;22	191	.001	<.001	FP (R); F2 (R)
36	-30;34;38	145	.005	.001	F2 (L); FP (L)
37	-40;-6;56	86	.036	.005	PRG (L)
Seed: Rostral prefrontal cortex (L)					
38	-42;-2;10	559	<.001	<.001	INS (L); CO (L); FO (L); F3o (L); PUT (L); PRG (L)
Seed: Rostral prefrontal cortex (R)					
39	10;48;36	1098	<.001	<.001	FP (L); FP (R); F1 (L); F1 (R); PAC (L); F2 (L); PAC (R)
40	-46;-58;-12	182	.006	<.001	TO2 (L); TO3 (L); OLi (L); TOF (L)
Seed: Supramarginal gyrus (L)					
41	64;-32;38	298	<.001	<.001	SGa (R); SGp (R)
42	-58;-30;46	146	.011	.001	SGa (L); POG (L)
43	26;-48;58	105	.031	.003	SPL (R)
44	-32;32;52	90	.041	.005	F2 (L)
45	-62;-64;6	83	.044	.007	TO2 (L); OLi (L)
Seed: Supramarginal gyrus (R)					
46	4;2;58	472	<.001	<.001	JPL (R); JPL (L); CGa; PAC (R); PAC (L)
47	54;-50;10	134	.032	.001	TO2 (R); AG (R)

Abbreviations: MNI: Montreal Neurological Institute, FDR: false discovery rate, unc: uncorrected, L: left, R: right, AG: angular gyrus, AMYG: amygdala, BS: brain-stem, CAU: caudate, CALC: intracalcarine cortex, CGa: cingulate gyrus, anterior division, CGp: cingulate gyrus, posterior division, CN: cuneal cortex, CO: central opercular cortex, CR: cerebellum, CRcr: cerebellum crus, F1: superior frontal gyrus, F2: middle frontal gyrus, F3o: inferior frontal gyrus, pars opercularis, F3t: inferior frontal gyrus, pars triangularis, FMC: frontal medial cortex, FO: frontal operculum cortex, FOC: frontal orbital cortex, FP: frontal pole, H: Heschl's gyrus, HP: hippocampus, INS: insular cortex, JPL: juxtapositional lobule cortex, LG: lingual gyrus, OF: occipital fusiform gyrus, OLi: lateral occipital cortex, inferior division, OLs: lateral occipital cortex, superior division, OP: occipital pole, PAL: pallidum, PHa: parahippocampal gyrus, anterior division, PHp: parahippocampal gyrus, posterior division, PO: parietal operculum cortex, POG: postcentral gyrus, PP: planum polare, PRG: precentral gyrus, PT: planum temporale, PUT: putamen, SC: subcallosal cortex, SCLC: supracalcarine cortex, SGa: supramarginal gyrus, anterior division, SGp: supramarginal gyrus, posterior division, SPL: superior parietal lobule, T1a: superior temporal gyrus, anterior division, T1p: superior temporal gyrus, posterior division, T2a: middle temporal gyrus, anterior division, T2p: middle temporal gyrus, posterior division, T3a: inferior temporal gyrus, anterior division, T3p: inferior temporal gyrus, posterior division, TFa: temporal fusiform cortex, anterior division, TFp: temporal fusiform cortex, posterior division, THL: thalamus, TO2: middle temporal gyrus, temporooccipital part, TO3: inferior temporal gyrus, temporooccipital part, TOF: temporal occipital fusiform cortex, TP: temporal pole, VM: vermis

Table S5. Clusters of voxels functionally connected with seeds of the sensorimotor, default mode and salience networks for the patients with a right hemispheric metastasis and their matched controls for the paradigm of the left hand

Cluster	MNI (x;y;z)	size	size p-FDR	size p-unc	Regions
**Network: Sensorimotor network**					
Seed: Lateral (R)					
1	18;-70;-50	262	.002	<.001	CR 8 (R); CR 7b (R)
2	-42;-66;8	133	.041	.001	OLi (L); TO2 (L)
Seed: Superior					

3	0;-16;38	275	.001	<.001	CGp; CGa; JPL (L); PRG (R)
4	-24;-58;-56	171	.007	<.001	CR 8 (L); CR 7b (L)
**Network: Default mode network**					
Seed: Medial prefrontal cortex					
5	-12;-88;-36	289	.001	<.001	CRcr 2 (L); CRcr 1 (L)
6	2;-56;-36	155	.016	.001	VM 9; CR 8 (L); CR 9 (R); CR 9 (L); CR 8 (R); VM 8
7	0;64;16	107	.049	.003	FP (L); FP (R)
8	-36;14;-30	103	.049	.003	TP (L); FOC (L)
**Network: Salience network**					
Seed: Anterior Insula (L)					
9	60;-40;38	333	<.001	<.001	SGp (R); AG (R); PT (R)
10	42;4;48	276	<.001	<.001	F2 (R); PRG (R)
11	2;-34;48	222	.001	<.001	PRG (R); CGp; POG (R); PCN; PRG (L)
12	36;-58;-46	192	.002	<.001	CR 8 (R); CR 7b (R); CRcr 1 (R)
13	8;-84;16	123	.019	.001	CALC (R); CN (R); SCLC (R); OP (R)
14	-36;38;20	97	.040	.003	FP (L); F2 (L); F3t (L)
15	6;24;50	95	.040	.003	PAC (R); PAC (L); F1 (R)
16	18;-76;-50	87	.048	.005	CR 8 (R); CR 7b (R)
Seed: Anterior Insula (R)					
17	-38;-64;-48	216	.004	<.001	CR 8 (L); CR 7b (L); CRcr 2 (L)
18	-42;-46;38	150	.016	.001	SGa (L); SGp (L)
19	-34;-66;-20	110	.044	.002	OF (L); CR 6 (L); CRcr 1 (L); TOF (L)
Seed: Rostral prefrontal cortex (L)					
20	-16;-78;38	156	.025	<.001	OLs (L); CN (L); PCN
21	-2;52;28	118	.045	.002	F1 (L); FP (L); PAC (L)
Seed: Supramarginal gyrus (L)					
22	-10;0;58	232	.003	<.001	F2 (L); F1 (L); PRG (L); JPL (L)
23	18;-74;-46	177	.009	<.001	CR 8 (R); CR 7b (R)
Seed: Supramarginal gyrus (R)					
24	16;-68;-48	820	<.001	<.001	CR 8 (R); CR 7b (R); CR 10 (R); VM 8; CRcr 2 (R); CR 9 (R); CR 6 (R)
25	-36;-64;-52	667	<.001	<.001	CR 8 (L); CR 7b (L); CRcr 2 (L); CRcr 1 (L)
26	-44;4;-6	117	.035	.002	INS (L); TP (L); CO (L); PP (L); FO (L)
27	40;-46;-12	109	.035	.003	TOF (R); CRcr 1 (R); CR 6 (R); TFp (R)

Abbreviations: MNI: Montreal Neurological Institute, FDR: false discovery rate, unc: uncorrected, L: left, R: right, AG: angular gyrus, AMYG: amygdala, BS: brain-stem, CAU: caudate, CALC: intracalcarine cortex, CGa: cingulate gyrus, anterior division, CGp: cingulate gyrus, posterior division, CN: cuneal cortex, CO: central opercular cortex, CR: cerebellum, CRcr: cerebellum crus, F1: superior frontal gyrus, F2: middle frontal gyrus, F3o: inferior frontal gyrus, pars opercularis, F3t: inferior frontal gyrus, pars triangularis, FMC: frontal medial cortex, FO: frontal operculum cortex, FOC: frontal orbital cortex, FP: frontal pole, H: Heschl's gyrus, HP: hippocampus, INS: insular cortex, JPL: juxtapositional lobule cortex, LG: lingual gyrus, OF: occipital fusiform gyrus, OLi: lateral occipital cortex, inferior division, OLs: lateral occipital cortex, superior division, OP: occipital pole, PAL: pallidum, PHa: parahippocampal gyrus, anterior division, PHp: parahippocampal gyrus, posterior division, PO: parietal operculum cortex, POG: postcentral gyrus, PP: planum polare, PRG: precentral gyrus, PT: planum temporale, PUT: putamen, SC: subcallosal cortex, SCLC: supracalcarine cortex, SGa: supramarginal gyrus, anterior division, SGp: supramarginal gyrus, posterior division, SPL: superior parietal lobule, T1a: superior temporal gyrus, anterior division, T1p: superior temporal gyrus, posterior division, T2a: middle temporal gyrus, anterior division, T2p: middle temporal gyrus, posterior division, T3a: inferior temporal gyrus, anterior division, T3p: inferior temporal gyrus, posterior division, TFa: temporal fusiform cortex, anterior division, TFp: temporal fusiform cortex, posterior division, THL: thalamus, TO2: middle temporal gyrus,

temporooccipital part, TO3: inferior temporal gyrus, temporooccipital part, TOF: temporal occipital fusiform cortex, TP: temporal pole, VM: vermis

Table S6. Clusters of voxels functionally connected with seeds of the sensorimotor, default mode and salience networks for the patients with a right hemispheric metastasis and their matched controls for the paradigm of the right hand

Cluster	MNI (x;y;z)	size	size p-FDR	size p-unc	Regions
**Network: Sensorimotor network**					
Seed: Lateral (L)					
1	56;-10;32	1110	<.001	<.001	POG (R); PRG (R); SGa (R); SGp (R); PO (R); PT (R); CO (R); AG (R)
2	-46;-4;36	590	<.001	<.001	PRG (L); POG (L); SGa (L)
3	-30;-68;-18	538	<.001	<.001	OLi (L); OF (L); CR 6 (L); TOF (L); CRcr 1 (L)
4	4;0;64	342	<.001	<.001	JPL (R); F1 (L); JPL (L); PAC (R); CGa; F1 (R)
5	-18;-64;-50	330	<.001	<.001	CR 8 (L); CR 7b (L)
6	44;0;8	246	<.001	<.001	CO (R); FO (R); INS (R); F3o (R); PRG (R)
7	18;-66;-48	190	.001	<.001	CR 8 (R); CR 7b (R)
8	-68;-42;20	111	.015	.002	SGp (L); PT (L); PO (L); T1p (L)
9	18;70;12	95	.025	.003	FP (R)
10	-38;-12;2	80	.042	.006	INS (L); CO (L); PP (L)
Seed: Lateral (R)					
11	-54;-14;36	976	<.001	<.001	POG (L); SGa (L); PO (L); SGp (L); PT (L); CO (L); H (L); PRG (L); T1p (L)
12	-24;-62;-54	548	<.001	<.001	CR 8 (L); CR 7b (L)
13	-22;-50;-6	466	<.001	<.001	OF (L); CR 6 (L); TOF (L); LG (L); OLi (L); CRcr 1 (L)
14	60;-30;34	377	<.001	<.001	SGa (R); POG (R); PO (R); CO (R); PT (R); PRG (R)
15	14;-74;-48	354	<.001	<.001	CR 8 (R); CR 7b (R); CRcr 2 (R)
16	-50;-78;-4	272	<.001	<.001	OLi (L); TO3 (L); TO2 (L)
17	50;-70;-6	154	.005	<.001	OLi (R); OF (R)
18	10;-28;-30	125	.012	.001	BS
19	14;4;72	121	.012	.001	JPL (R); F1 (R)
20	52;10;4	115	.014	.002	F3o (R); CO (R); INS (R); FO (R); PRG (R); TP (R)
21	54;-66;6	110	.015	.002	OLi (R); TO2 (R)
22	-50;2;14	88	.033	.005	PRG (L); CO (L)
23	34;22;14	80	.043	.006	FO (R); INS (R)
Seed: Superior					
24	58;-22;22	913	<.001	<.001	SGa (R); PO (R); POG (R); SGp (R); CO (R); PT (R)
25	-24;-62;-56	467	<.001	<.001	CR 8 (L); CR 7b (L)
26	48;10;-2	414	<.001	<.001	F3o (R); CO (R); FO (R); PRG (R); INS (R); TP (R)
27	-42;-6;4	317	<.001	<.001	CO (L); INS (L); FO (L); PP (L); PRG (L)
28	30;-62;-54	279	<.001	<.001	CR 8 (R); CR 9 (R)
29	-32;-68;-18	237	<.001	<.001	OF (L); CR 6 (L); TOF (L); OLi (L); CRcr 1 (L)
30	36;-54;-22	206	.001	<.001	CR 6 (R); TOF (R); CR 4 5 (R); TO3 (R); OF (R); CRcr 1 (R)
31	-56;-26;44	112	.016	.002	SGa (L); POG (L)
32	-46;-30;16	111	.016	.002	PO (L); SGa (L); CO (L)
33	-38;-46;-30	101	.020	.002	CRcr 1 (L); CR 6 (L); TFp (L)
34	-50;-62;8	100	.020	.003	TO2 (L); OLi (L)

35	-40;-72;-38	90	.027	.004	CRcr 2 (L); CRcr 1 (L)
36	44;-56;6	85	.031	.005	TO2 (R); OLi (R)
37	-10;-88;-28	73	.048	.008	CRcr 2 (L)
**Network: Default mode network**					
Seed: Medial prefrontal cortex					
38	20;42;-18	242	.001	<.001	FP (R); FOC (R); FMC
39	34;-62;-52	237	.001	<.001	CR 8 (R)
40	-24;-54;72	153	.009	<.001	SPL (L); POG (L); OLs (L)
41	-52;-12;-18	146	.009	<.001	T2p (L); T2a (L); T1p (L)
42	-46;6;-4	131	.010	.001	INS (L); CO (L); PP (L); TP (L); FO (L)
43	24;-30;-14	128	.010	.001	PHp (R); HP (R); BS; CR 3 (R)
44	-26;-24;-14	126	.010	.001	HP (L); PHp (L)
45	52;-58;26	118	.012	.001	AG (R); OLs (R)
46	-20;-76;-54	109	.015	.002	
47	-40;-50;-46	101	.017	.003	CRcr 2 (L); CR 7b (L); CR 8 (L); CRcr 1 (L)
48	-8;68;22	100	.017	.003	FP (L)
49	24;-88;-38	99	.017	.003	CRcr 2 (R)
50	-14;-90;-36	90	.022	.004	CRcr 2 (L); CRcr 1 (L)
51	38;16;-4	75	.039	.007	INS (R); FOC (R); FO (R)
52	42;-78;-38	71	.043	.009	CRcr 2 (R); CRcr 1 (R)
Seed: Lateral parietal (L)					
53	20;-26;-16	350	<.001	<.001	PHp (R); BS; HP (R); LG (R); TOF (R); CR 3 (R); TFp (R); VM 1 2
54	-60;-6;-20	280	<.001	<.001	T2a (L); T2p (L); T3p (L); T3a (L)
55	-18;-14;-18	261	<.001	<.001	HP (L); PHp (L); PHa (L); AMYG (L); BS
56	-18;36;-12	120	.026	.002	FOC (L); FMC; FP (L)
57	-24;-42;-16	109	.027	.002	TFp (L); PHp (L); CR 4 5 (L); LG (L); HP (L); TOF (L)
58	42;-72;-40	108	.027	.002	CRcr 2 (R); CRcr 1 (R)
Seed: Lateral parietal (R)					
59	-18;-30;-16	353	<.001	<.001	PHp (L); CR 4 5 (L); HP (L); LG (L); TFp (L); TOF (L); CR 3 (L)
60	18;-26;-18	336	<.001	<.001	PHp (R); CR 3 (R); HP (R); BS; LG (R); TOF (R); TFp (R); CR 4 5 (R)
61	14;-56;26	123	.025	.001	PCN
62	60;-12;-14	114	.026	.002	T2p (R); T2a (R); T1a (R)
Seed: Posterior cingulate cortex					
63	24;-38;-12	174	.009	<.001	PHp (R); CR 3 (R); LG (R); TFp (R); TOF (R); BS
**Network: Salience network**					
Seed: Anterior cingulate cortex					
64	-52;0;-2	299	<.001	<.001	PP (L); INS (L); CO (L); TP (L); PRG (L); T1a (L)
Seed: Anterior Insula (L)					
65	-16;-10;64	545	<.001	<.001	JPL (R); JPL (L); F1 (L); F1 (R); PRG (L)
66	-24;-62;-50	294	<.001	<.001	CR 8 (L); CR 7b (L); CRcr 2 (L)
67	-54;-36;36	170	.005	<.001	SGa (L); SGp (L); PO (L)
68	-40;38;32	169	.005	<.001	FP (L); F2 (L)
69	8;-92;-30	134	.012	.001	CRcr 2 (R); CRcr 2 (L)
70	-30;-60;-24	126	.014	.001	CR 6 (L); TOF (L); LG (L); OF (L)

Seed: Anterior Insula (R)					
71	-56;-8;36	2466	<.001	<.001	POG (L); PRG (L); SGa (L); PO (L); SGp (L); SPL (L); PT (L); F2 (L); F1 (L); T1p (L)
72	12;4;48	1626	<.001	<.001	JPL (R); F1 (R); JPL (L); F1 (L); CGa; PAC (R); PAC (L); PRG (L); PRG (R)
73	-14;-40;62	356	<.001	<.001	POG (L); SPL (L); PRG (L)
74	34;-28;60	208	.001	<.001	POG (R); PRG (R); SPL (R)
75	44;-58;8	158	.006	<.001	OLi (R); TO2 (R)
76	-28;-92;4	135	.011	.001	OP (L); OLi (L)
77	-6;-90;-36	126	.013	.001	CRcr 2 (L); CRcr 2 (R)
78	40;-56;-18	120	.014	.001	TOF (R); OF (R); CR 6 (R); TO3 (R)
79	54;-34;46	114	.015	.002	SGa (R); SGp (R)
80	56;-42;16	102	.020	.002	SGp (R); TO2 (R); AG (R); PT (R)
81	-32;-60;-24	102	.020	.002	CR 6 (L); TOF (L); CRcr 1 (L)
82	-54;6;-6	97	.022	.003	TP (L); PP (L); CO (L); PRG (L)
83	62;2;40	93	.024	.004	PRG (R)
84	-46;-66;4	75	.048	.008	OLi (L); TO2 (L)
Seed: Rostral prefrontal cortex (L)					
85	6;68;22	1301	<.001	<.001	FP (L); FP (R); PAC (L); F1 (L); PAC (R); F1 (R); CGa
86	-52;4;6	371	<.001	<.001	CO (L); PRG (L); FO (L); F3o (L); INS (L); TP (L); PP (L)
87	-58;-34;34	331	<.001	<.001	SGa (L); PO (L); SGp (L); POG (L)
88	-36;-86;-10	276	<.001	<.001	OLi (L); OP (L)
89	34;-40;44	219	.001	<.001	SPL (R); SGp (R); POG (R)
90	-22;-76;-12	215	.001	<.001	OF (L); CR 6 (L); TOF (L); OP (L)
91	56;-24;24	145	.006	.001	PO (R); SGa (R); POG (R)
92	24;-72;-58	141	.006	.001	CR 8 (R); CR 7b (R); CRcr 2 (R)
93	-36;-44;44	114	.013	.002	SPL (L); SGp (L); SGa (L)
Seed: Rostral prefrontal cortex (R)					
94	12;-90;-40	276	<.001	<.001	CRcr 2 (R); CRcr 1 (R); CRcr 2 (L)
95	-44;-40;40	276	<.001	<.001	SGa (L); POG (L); SGp (L); SPL (L)
96	10;42;16	200	.001	<.001	PAC (L); PAC (R); CGa; F1 (L)
97	-44;6;-2	200	.001	<.001	CO (L); INS (L); TP (L); PRG (L); PP (L); FO (L)
98	34;-42;46	119	.017	.001	SPL (R); POG (R); SGp (R)
99	34;14;12	111	.017	.002	FO (R); INS (R); CO (R); PUT (R)
100	-12;68;0	109	.017	.002	FP (L); FP (R)
101	-4;-4;60	82	.045	.006	JPL (R); JPL (L); F1 (R)
Seed: Supramarginal gyrus (L)					
102	-52;2;24	4080	<.001	<.001	PRG (L); JPL (R); JPL (L); F1 (R); PRG (R); F1 (L); INS (L); CGa; CO (L); PAC (L); FO (L); PAC (R); F3o (L); TP (L); PP (L); F2 (L)
103	-42;-54;-20	1008	<.001	<.001	OLi (L); CRcr 1 (L); TOF (L); CR 6 (L); TO3 (L); TO2 (L); OF (L); TFp (L)
104	56;0;16	879	<.001	<.001	PRG (R); INS (R); CO (R); POG (R); F3o (R); FO (R); F2 (R); TP (R); PP (R)
105	-26;-54;-48	406	<.001	<.001	CR 8 (L); CR 7b (L); CRcr 2 (L)
106	-38;36;26	324	<.001	<.001	F2 (L); FP (L)
107	-12;-88;-34	223	.001	<.001	CRcr 2 (L); LG (L); CRcr 2 (R); VM 7

108	-52;-34;50	176	.002	<.001	POG (L); SGa (L); SPL (L); SGp (L)
109	18;-64;-48	161	.003	<.001	CR 8 (R); CR 7b (R); CR 9 (R); CRcr 2 (R)
110	-26;-50;28	98	.026	.003	
Seed: Supramarginal gyrus (R)					
111	8;2;64	3763	<.001	<.001	PRG (R); POG (L); PRG (L); JPL (R); F1 (R); JPL (L); F1 (L); SPL (L); CGa; F2 (R); F3o (R); PAC (R)
112	2;-84;-28	478	<.001	<.001	CRcr 2 (L); CRcr 2 (R); CRcr 1 (R); LG (L); CRcr 1 (L); VM 7
113	52;10;2	380	<.001	<.001	CO (R); PRG (R); FO (R); F3o (R); INS (R); TP (R); PUT (R)
114	-50;-70;-10	279	<.001	<.001	OLi (L); TO2 (L); TO3 (L); OF (L)
115	-46;2;2	222	.001	<.001	INS (L); CO (L); PP (L); TP (L); FO (L)
116	34;-28;58	198	.001	<.001	POG (R); SPL (R); PRG (R)
117	-44;-46;-32	193	.001	<.001	CRcr 1 (L); CR 6 (L); TOF (L); TO3 (L); TFp (L)
118	-52;-4;36	143	.006	.001	PRG (L); POG (L)
119	-22;-62;-52	143	.006	.001	CR 8 (L)
120	60;-44;18	133	.007	.001	SGp (R); AG (R); TO2 (R); PO (R); PT (R)
121	56;-8;-40	110	.015	.002	T3a (R); T3p (R)
122	24;-62;-52	95	.024	.004	CR 8 (R)
123	18;-48;-28	92	.025	.004	CR 6 (R); CR 4 5 (R)
124	44;-68;-8	75	.047	.008	OLi (R); OF (R)

Abbreviations: MNI: Montreal Neurological Institute, FDR: false discovery rate, unc: uncorrected, L: left, R: right, AG: angular gyrus, AMYG: amygdala, BS: brain-stem, CAU: caudate, CALC: intracalcarine cortex, CGa: cingulate gyrus, anterior division, CGp: cingulate gyrus, posterior division, CN: cuneal cortex, CO: central opercular cortex, CR: cerebellum, CRcr: cerebellum crus, F1: superior frontal gyrus, F2: middle frontal gyrus, F3o: inferior frontal gyrus, pars opercularis, F3t: inferior frontal gyrus, pars triangularis, FMC: frontal medial cortex, FO: frontal operculum cortex, FOC: frontal orbital cortex, FP: frontal pole, H: Heschl's gyrus, HP: hippocampus, INS: insular cortex, JPL: juxtapositional lobule cortex, LG: lingual gyrus, OF: occipital fusiform gyrus, OLi: lateral occipital cortex, inferior division, OLs: lateral occipital cortex, superior division, OP: occipital pole, PAL: pallidum, PHa: parahippocampal gyrus, anterior division, PHp: parahippocampal gyrus, posterior division, PO: parietal operculum cortex, POG: postcentral gyrus, PP: planum polare, PRG: precentral gyrus, PT: planum temporale, PUT: putamen, SC: subcallosal cortex, SCLC: supracalcarine cortex, SGa: supramarginal gyrus, anterior division, SGp: supramarginal gyrus, posterior division, SPL: superior parietal lobule, T1a: superior temporal gyrus, anterior division, T1p: superior temporal gyrus, posterior division, T2a: middle temporal gyrus, anterior division, T2p: middle temporal gyrus, posterior division, T3a: inferior temporal gyrus, anterior division, T3p: inferior temporal gyrus, posterior division, TFa: temporal fusiform cortex, anterior division, TFp: temporal fusiform cortex, posterior division, THL: thalamus, TO2: middle temporal gyrus, temporooccipital part, TO3: inferior temporal gyrus, temporooccipital part, TOF: temporal occipital fusiform cortex, TP: temporal pole, VM: vermis

Table S7. Clusters of voxels functionally connected with seeds of the sensorimotor, default mode and salience networks for the patients with a right hemispheric metastasis and their matched controls for the paradigm of the left foot

Cluster	MNI (x;y;z)	size	size p-FDR	size p-unc	Regions
**Network: Sensorimotor network**					
Seed: Lateral (L)					
1	50;8;18	779	<.001	<.001	PRG (R); POG (R); CO (R); F3o (R); SGa (R); PO (R); PP (R); FO (R); PT (R)
2	-40;-52;-30	423	<.001	<.001	TOF (L); CRcr 1 (L); CR 6 (L); TO3 (L); TFp (L); OF (L)
3	-48;-10;44	307	<.001	<.001	PRG (L); POG (L)
4	38;-4;64	209	.001	<.001	PRG (R); F1 (R); F2 (R)
5	-14;-72;-48	154	.005	<.001	CR 8 (L); CR 7b (L)
6	12;2;60	144	.006	<.001	CGa; JPL (R); PAC (R); F1 (R)
7	-62;-22;22	108	.017	.002	POG (L); SGa (L); CO (L); PO (L)

8	32;-54;-28	108	.017	.002	CR 6 (R); TOF (R)
9	54;-50;-16	89	.033	.003	TO3 (R); OLi (R); TO2 (R)
Seed: Lateral (R)					
10	-58;-18;20	1032	<.001	<.001	POG (L); PO (L); SGa (L); CO (L); PRG (L); PT (L); SPL (L); H (L); SGp (L)
11	-4;64;14	743	<.001	<.001	FP (L); FP (R); FMC; PAC (R)
12	-48;-48;-22	611	<.001	<.001	CR 6 (L); TO3 (L); TOF (L); CRcr 1 (L); OF (L); T3p (L); TFp (L); CR 4 5 (L)
13	-38;-8;54	240	<.001	<.001	PRG (L); F2 (L); F1 (L)
14	-10;-76;-48	189	.001	<.001	CR 8 (L); CR 7b (L); CRcr 2 (L)
15	-52;-72;4	162	.003	<.001	OLi (L); TO2 (L)
16	-20;-52;74	139	.006	<.001	SPL (L); POG (L); OLs (L)
17	28;-44;70	129	.008	.001	SPL (R); POG (R)
18	44;-42;-16	123	.008	.001	TO3 (R); TOF (R); TO2 (R)
19	-12;-60;56	120	.009	.001	OLs (L); SPL (L); PCN
20	-42;4;18	91	.025	.003	PRG (L); F3o (L); CO (L)
21	-8;-16;-8	77	.042	.006	THL (L)
22	-32;34;22	73	.047	.007	FP (L); F2 (L)
Seed: Superior					
23	-36;-56;-24	368	<.001	<.001	CR 6 (L); TOF (L); CRcr 1 (L)
24	-32;-6;6	283	<.001	<.001	INS (L); PUT (L); CO (L); F3o (L); FO (L); PRG (L)
25	32;10;8	276	<.001	<.001	CO (R); INS (R); PRG (R); FO (R); F3o (R); PUT (R)
26	-62;-32;30	262	<.001	<.001	SGa (L); PO (L); POG (L); CO (L); PT (L)
27	4;64;26	224	<.001	<.001	FP (L); FP (R)
28	-20;-72;-50	198	.001	<.001	CR 8 (L); CR 7b (L)
29	-4;-18;-6	162	.002	<.001	THL (L)
30	62;-30;22	158	.003	<.001	PO (R); SGa (R); PT (R); SGp (R)
31	-44;6;18	128	.007	.001	F3o (L); PRG (L)
32	-34;34;20	122	.007	.001	F2 (L); FP (L)
33	14;-60;-52	107	.012	.002	CR 8 (R); CR 7b (R); CR 9 (R)
34	-38;-76;-36	105	.012	.002	CRcr 2 (L); CRcr 1 (L)
35	14;-86;-40	92	.019	.003	CRcr 2 (R)
36	12;-52;64	69	.047	.009	SPL (R); PCN
**Network: Default mode network**					
Seed: Medial prefrontal cortex					
37	60;2;30	571	<.001	<.001	PRG (R); POG (R); CO (R); F3o (R)
38	-58;4;18	309	<.001	<.001	PRG (L); F3o (L)
39	-34;-4;56	297	<.001	<.001	PRG (L); F2 (L); F1 (L); POG (L)
40	-46;28;28	140	.008	<.001	F2 (L); FP (L); F3t (L)
41	-50;36;8	122	.013	.001	F3t (L); FP (L)
42	52;44;10	115	.014	.001	FP (R)
43	-46;-10;36	97	.025	.002	PRG (L); POG (L)
Seed: Lateral parietal (R)					
44	36;-12;-32	161	.013	<.001	TFp (R); T3p (R); PHa (R)
**Network: Salience network**					

Seed: Anterior cingulate cortex					
45	-26;-78;-14	516	<.001	<.001	OLi (L); OF (L); TO2 (L); TO3 (L)
46	40;-44;-20	312	<.001	<.001	TOF (R); CR 6 (R); OF (R); TO3 (R); CRcr 1 (R)
47	-50;-20;6	287	<.001	<.001	H (L); CO (L); PT (L); INS (L); POG (L)
48	-42;10;-6	260	<.001	<.001	INS (L); CO (L); PP (L); TP (L); FO (L); PRG (L)
49	-20;-66;-48	211	.001	<.001	CR 8 (L); CR 7b (L); CRcr 2 (L)
50	-2;-26;-4	102	.025	.002	THL (L); BS
Seed: Anterior Insula (L)					
51	-56;-36;44	298	<.001	<.001	SGa (L); SGp (L); POG (L); SPL (L)
52	-18;-62;48	281	<.001	<.001	SPL (L); OLs (L); POG (L)
53	20;-26;62	274	<.001	<.001	PRG (R); F2 (R); POG (R)
54	42;-76;2	180	.002	<.001	OLi (R); TO2 (R); OLs (R)
55	24;-68;-24	178	.002	<.001	CR 6 (R); OF (R); TOF (R); CRcr 1 (R)
56	10;10;72	148	.004	<.001	F1 (R)
57	-28;-16;66	141	.004	<.001	PRG (L); F1 (L)
58	24;-84;-4	128	.006	.001	OF (R); OP (R); LG (R)
59	22;-70;-50	112	.011	.001	CR 8 (R); CR 7b (R)
60	18;46;24	101	.015	.002	FP (R)
61	-52;-4;44	69	.049	.008	PRG (L)
62	48;-30;46	69	.049	.008	SGa (R); POG (R); SGp (R)
63	4;-18;54	68	.049	.008	PRG (R); JPL (L)
Seed: Anterior Insula (R)					
64	36;-24;48	300	<.001	<.001	POG (R); SPL (R); PRG (R); SGa (R); SGp (R)
65	44;-42;-18	155	.008	<.001	CR 6 (R); TOF (R); CRcr 1 (R)
66	-8;0;68	137	.009	<.001	F1 (L); JPL (L)
67	-44;-40;36	130	.009	.001	SGa (L); SGp (L)
68	-46;8;18	129	.009	.001	F3o (L); PRG (L)
69	-48;36;10	113	.013	.001	FP (L); F3t (L)
70	10;12;60	111	.013	.001	JPL (R); F1 (R)
71	-28;-14;70	106	.014	.001	PRG (L)
72	-16;-58;54	103	.014	.002	OLs (L); SPL (L)
73	-48;-6;42	87	.025	.003	PRG (L)
74	-12;-44;66	81	.029	.004	POG (L)
75	-44;-10;54	73	.039	.006	PRG (L); POG (L)
76	16;-26;64	70	.041	.007	PRG (R); POG (R)
Seed: Rostral prefrontal cortex (L)					
77	-22;-88;-16	1315	<.001	<.001	OLi (L); OF (L); OP (L); TO3 (L); OLs (L); TO2 (L); TOF (L)
78	32;-76;-12	735	<.001	<.001	OF (R); OLi (R); CR 6 (R); TOF (R); OLs (R)
79	12;-80;-2	489	<.001	<.001	CALC (R); LG (R); LG (L); CALC (L); OF (R); OP (R); CR 6 (L); CR 6 (R); SCLC (R)
80	-48;-54;-24	294	<.001	<.001	TOF (L); CR 6 (L); TO3 (L); CRcr 1 (L); TFp (L); CR 4 5 (L)
81	38;-34;-30	151	.004	<.001	TFp (R); TOF (R); CR 6 (R); CRcr 1 (R)
82	-32;38;20	119	.010	.001	FP (L); F2 (L)
83	-20;-82;-48	118	.010	.001	CR 8 (L); CRcr 2 (L); CR 7b (L)

84	-20;-60;44	113	.011	.001	OLs (L); SPL (L)
85	44;-62;10	106	.013	.002	OLi (R); TO2 (R); AG (R); OLs (R)
86	-16;32;52	100	.015	.002	F1 (L); FP (L)
87	-38;4;-6	98	.015	.002	INS (L); TP (L); FO (L); CO (L)
88	52;14;18	71	.044	.007	F3o (R); PRG (R)
Seed: Rostral prefrontal cortex (R)					
89	6;-58;4	632	<.001	<.001	LG (R); CALC (R); LG (L); CALC (L); SCLC (R); VM 4 5; OF (R)
90	-38;-86;-8	363	<.001	<.001	OLi (L); OP (L); OF (L)
91	-20;-76;-50	120	.023	.001	CR 8 (L); CR 7b (L); CRcr 2 (L)
92	60;-58;-4	117	.023	.001	TO2 (R); TO3 (R); OLi (R)
93	-38;36;22	105	.027	.002	F2 (L); FP (L)
94	-50;-52;-20	103	.027	.002	TO3 (L); TOF (L)
Seed: Supramarginal gyrus (L)					
95	-56;-6;50	399	<.001	<.001	PRG (L); POG (L)
96	-40;-50;-20	271	<.001	<.001	TOF (L); TFp (L); TO3 (L); CR 6 (L); CRcr 1 (L)
97	-52;-64;0	212	.001	<.001	OLi (L); TO2 (L); OF (L); TO3 (L)
98	56;-52;-6	141	.006	<.001	TO2 (R); OLi (R); TO3 (R)
99	42;8;8	139	.006	<.001	PRG (R); CO (R); INS (R); F3o (R); FO (R)
100	-38;-48;68	134	.006	<.001	POG (L); SPL (L)
101	-46;2;18	133	.006	<.001	PRG (L); F3o (L)
102	38;-4;60	118	.010	.001	PRG (R); F2 (R); F1 (R)
103	-28;-16;68	111	.012	.001	PRG (L); F1 (L)
104	18;6;66	107	.013	.001	F1 (R)
105	-16;-76;-42	100	.015	.002	CR 7b (L); CRcr 2 (L); CR 8 (L)
106	52;4;-6	89	.022	.003	PP (R); TP (R); T1a (R); INS (R)
107	-54;-38;40	86	.024	.003	SGa (L); SGp (L)
108	-30;-44;42	80	.029	.004	SPL (L)
109	30;-52;-32	72	.039	.006	CR 6 (R)
110	-62;-36;14	69	.042	.007	PT (L); T1p (L); PO (L); SGp (L)
111	22;-74;-50	68	.042	.007	CR 7b (R); CR 8 (R)
112	8;72;8	65	.046	.008	FP (R); FP (L)
Seed: Supramarginal gyrus (R)					
113	-46;6;20	251	.001	<.001	PRG (L); F3o (L)
114	26;-40;22	219	.001	<.001	
115	-60;-32;24	140	.009	<.001	PO (L); PT (L); SGa (L); SGp (L); CO (L)
116	62;-64;2	139	.009	<.001	OLi (R); TO2 (R); TO3 (R)
117	-22;-68;-50	130	.010	.001	CR 8 (L); CR 7b (L); CRcr 2 (L)

Abbreviations: MNI: Montreal Neurological Institute, FDR: false discovery rate, unc: uncorrected, L: left, R: right, AG: angular gyrus, AMYG: amygdala, BS: brain-stem, CAU: caudate, CALC: intracalcarine cortex, CGa: cingulate gyrus, anterior division, CGp: cingulate gyrus, posterior division, CN: cuneal cortex, CO: central opercular cortex, CR: cerebellum, CRcr: cerebellum crus, F1: superior frontal gyrus, F2: middle frontal gyrus, F3o: inferior frontal gyrus, pars opercularis, F3t: inferior frontal gyrus, pars triangularis, FMC: frontal medial cortex, FO: frontal operculum cortex, FOC: frontal orbital cortex, FP: frontal pole, H: Heschl's gyrus, HP: hippocampus, INS: insular cortex, JPL: juxtapositional lobule cortex, LG: lingual gyrus, OF: occipital fusiform gyrus, OLi: lateral occipital cortex, inferior division, OLs: lateral occipital cortex, superior division, OP: occipital pole, PAL: pallidum, PHa: parahippocampal gyrus, anterior division, PHp: parahippocampal gyrus, posterior division, PO: parietal operculum cortex, POG: postcentral gyrus, PP: planum polare, PRG: precentral gyrus, PT: planum temporale, PUT: putamen, SC: subcallosal cortex, SCLC: supracalcarine cortex, SGa: supramarginal

gyrus, anterior division, SGp: supramarginal gyrus, posterior division, SPL: superior parietal lobule, T1a: superior temporal gyrus, anterior division, T1p: superior temporal gyrus, posterior division, T2a: middle temporal gyrus, anterior division, T2p: middle temporal gyrus, posterior division, T3a: inferior temporal gyrus, anterior division, T3p: inferior temporal gyrus, posterior division, TFa: temporal fusiform cortex, anterior division, TFp: temporal fusiform cortex, posterior division, THL: thalamus, TO2: middle temporal gyrus, temporooccipital part, TO3: inferior temporal gyrus, temporooccipital part, TOF: temporal occipital fusiform cortex, TP: temporal pole, VM: vermis

Table S8. Clusters of voxels functionally connected with seeds of the sensorimotor, default mode and salience networks for the patients with a right hemispheric metastasis and their matched controls for the paradigm of the right foot

Cluster	MNI (x;y;z)	size	size p-FDR	size p-unc	Regions
**Network: Sensorimotor network**					
Seed: Lateral (L)					
1	-52;-14;40	174	.003	<.001	POG (L); PRG (L)
2	46;-2;4	166	.003	<.001	CO (R); INS (R); PP (R); H (R)
3	-64;-8;18	162	.003	<.001	CO (L); POG (L); PRG (L); PP (L); T1a (L)
4	60;-10;14	104	.019	.002	CO (R); POG (R); PT (R)
Seed: Lateral (R)					
5	-52;-24;26	709	<.001	<.001	CO (L); H (L); PP (L); POG (L); INS (L); PO (L); PT (L); TP (L); SGa (L); PRG (L); FO (L)
6	50;-42;12	279	<.001	<.001	SGp (R); TO2 (R); PT (R); AG (R)
7	-40;-66;-2	128	.018	.001	OLi (L); TO2 (L); TO3 (L)
8	32;-58;-50	115	.018	.001	CR 8 (R); CR 7b (R)
9	58;-10;16	113	.018	.001	CO (R); POG (R); PRG (R); PO (R)
10	-8;6;56	111	.018	.001	JPL (L); PAC (L); F1 (L)
11	-32;-6;50	89	.038	.004	PRG (L); F2 (L)
12	-34;-16;10	86	.038	.004	INS (L); PUT (L); CO (L)
Seed: Superior					
13	-10;34;60	1148	<.001	<.001	F1 (L); FP (L); FP (R); F1 (R)
14	-48;-48;-22	606	<.001	<.001	TO3 (L); CRcr 1 (L); TOF (L); CR 6 (L); T3p (L); TFp (L); OLi (L); CR 8 (L); CR 7b (L); CR 4 5 (L)
15	-38;-68;-12	178	.002	<.001	OLi (L); OF (L); CRcr 1 (L); CR 6 (L)
16	-46;4;-8	103	.019	.002	INS (L); PP (L); TP (L); CO (L)
17	-22;-58;-54	101	.019	.002	CR 8 (L); CR 9 (L)
**Network: Salience network**					
Seed: Anterior Insula (L)					
18	12;-30;54	211	.002	<.001	PRG (R); POG (R); PCN
19	-26;-42;14	196	.002	<.001	
20	-40;-48;-22	184	.002	<.001	TOF (L); CR 6 (L); TO3 (L); TFp (L); CRcr 1 (L)
21	32;-46;54	143	.007	<.001	SPL (R)
22	-14;-78;-44	133	.008	.001	CR 7b (L); VM 8; CRcr 2 (L); VM 7; CRcr 2 (R)
23	4;-2;48	124	.009	.001	JPL (R); CGa; JPL (L)
24	-28;-64;-52	89	.032	.003	CR 8 (L); CR 7b (L)
Seed: Anterior Insula (R)					
25	54;-6;50	249	.001	<.001	PRG (R); F2 (R); POG (R)
26	-50;-14;44	192	.002	<.001	PRG (L); POG (L); F2 (L)
27	12;-90;-34	130	.012	.001	CRcr 2 (R); CRcr 1 (R)
28	-8;0;56	116	.015	.001	JPL (L); F1 (L)

Seed: Rostral prefrontal cortex (L)					
29	-16;-64;10	816	<.001	<.001	CALC (L); LG (L); CALC (R); PCN; SCLC (R); OLs (R); SCLC (L); OP (L); OP (R); CR 6 (L); CN (L); CN (R); LG (R)
30	-46;22;34	225	.001	<.001	F2 (L); F3t (L); FP (L); F3o (L)
31	-6;46;20	147	.006	<.001	PAC (L); PAC (R); F1 (L)
32	-12;28;42	108	.020	.002	F1 (L); PAC (L); F2 (L)
Seed: Rostral prefrontal cortex (R)					
33	0;44;46	1577	<.001	<.001	F1 (L); FP (L); PAC (L); F2 (L); F1 (R); PAC (R); FP (R)
Seed: Supramarginal gyrus (L)					
34	-52;-36;-24	369	<.001	<.001	TO3 (L); TOF (L); T3p (L); CRcr 1 (L); TFp (L); CR 6 (L)
35	-38;-76;-6	293	<.001	<.001	OLi (L); OF (L); TO3 (L)
36	4;-76;-36	143	.007	<.001	CR 7b (L); CRcr 2 (R); CRcr 2 (L); VM 7; VM 8; CR 7b (R)
37	6;44;46	114	.016	.001	F1 (R); F1 (L); FP (L); FP (R)
38	-30;-60;-54	110	.016	.001	CR 8 (L)
Seed: Supramarginal gyrus (R)					
39	-16;-80;-48	528	<.001	<.001	CR 8 (L); CR 7b (L); CRcr 2 (L)
40	38;-10;70	195	.002	<.001	PRG (R); F1 (R); JPL (R); F2 (R)
41	-6;54;38	143	.009	<.001	F1 (L); FP (L); F1 (R)

Abbreviations: MNI: Montreal Neurological Institute, FDR: false discovery rate, unc: uncorrected, L: left, R: right, AG: angular gyrus, AMYG: amygdala, BS: brain-stem, CAU: caudate, CALC: intracalcarine cortex, CGa: cingulate gyrus, anterior division, CGp: cingulate gyrus, posterior division, CN: cuneal cortex, CO: central opercular cortex, CR: cerebellum, CRcr: cerebellum crus, F1: superior frontal gyrus, F2: middle frontal gyrus, F3o: inferior frontal gyrus, pars opercularis, F3t: inferior frontal gyrus, pars triangularis, FMC: frontal medial cortex, FO: frontal operculum cortex, FOC: frontal orbital cortex, FP: frontal pole, H: Heschl's gyrus, HP: hippocampus, INS: insular cortex, JPL: juxtapositional lobule cortex, LG: lingual gyrus, OF: occipital fusiform gyrus, OLi: lateral occipital cortex, inferior division, OLs: lateral occipital cortex, superior division, OP: occipital pole, PAL: pallidum, PHa: parahippocampal gyrus, anterior division, PHp: parahippocampal gyrus, posterior division, PO: parietal operculum cortex, POG: postcentral gyrus, PP: planum polare, PRG: precentral gyrus, PT: planum temporale, PUT: putamen, SC: subcallosal cortex, SCLC: supracalcarine cortex, SGa: supramarginal gyrus, anterior division, SGp: supramarginal gyrus, posterior division, SPL: superior parietal lobule, T1a: superior temporal gyrus, anterior division, T1p: superior temporal gyrus, posterior division, T2a: middle temporal gyrus, anterior division, T2p: middle temporal gyrus, posterior division, T3a: inferior temporal gyrus, anterior division, T3p: inferior temporal gyrus, posterior division, TFa: temporal fusiform cortex, anterior division, TFp: temporal fusiform cortex, posterior division, THL: thalamus, TO2: middle temporal gyrus, temporooccipital part, TO3: inferior temporal gyrus, temporooccipital part, TOF: temporal occipital fusiform cortex, TP: temporal pole, VM: vermis
